# Supplementary material for: Modelling Skylarks (Alauda arvensis) to Predict Impacts of Changes in Land Management and Policy: Development and Testing of an Agent-Based Model
Source: PLoS One. 2013 Jun 6;8(6):e65803. doi: 10.1371/journal.pone.0065803 (PMC3675089; doi:10.1371/journal.pone.0065803)
Supplement: Supporting Information S4 — The skylark ODdox as a zipped archive. (ZIP) [file pone.0065803.s004.zip › Skylark_ODdox/class_sk_territories.html]

ALMaSS Skylark ODdox: SkTerritories Class Reference


ALMaSS Skylark ODdox
 2.0


- Main Page
- Related Pages
- Classes
- Files

- Class List
- Class Index
- Class Hierarchy
- Class Members

Public Member Functions |
Public Attributes |
Private Member Functions |
Private Attributes

SkTerritories Class Reference

`#include <skylarks_all.h>`

List of all members.

|  |  |
| --- | --- |
| Public Member Functions | |
| void | ClaimGrid (int x, int y, int range) |
| void | EvaluateAllTerritories (void) |
| double | EvaluateHabitatN (skTTerritory \*a\_terr) |
| void | FemaleOccupy (int ref, Skylark\_Female \*Female) |
| void | GetTerritoriesByDistance (int nx, int ny, vector< Point > \*alist) |
| bool | IsExtGridPositionValid (int &x, int &y, int range) |
| bool | IsGridPositionValid (int &x, int &y, int range) |
| int | IsValid (int nx, int ny) |
| void | Occupy (int ref, Skylark\_Male \*Male) |
| void | PreCachePoly (int a\_poly) |
| double | PrePoly2Qual (int a\_poly) |
| double | PrePolyNQual (int a\_poly, int \*a\_good\_polys) |
| void | PreProcessLandscape2 (Landscape \*L) |
| void | RemoveFemale (int ref) |
| void | RemoveMale (int ref) |
|  | SkTerritories (Landscape \*L) |
| void | Split (int ref) |
| Skylark\_Female \* | Supply\_F\_Owner (int ref) |
| Skylark\_Male \* | Supply\_Owner (int ref) |
| int | Supply\_quality (int ref) |
| int | Supply\_size (int ref) |
| skTTerritory \* | Supply\_terr (int ref) |
| int | Supply\_x (int ref) |
| int | Supply\_y (int ref) |
| bool | SupplyIsNestValid (int ref) |
| Point | SupplyNestPosition (int ref) |
| int | SupplyNoFemaleOccupied () |
| int | SupplyNoMaleOccupied () |
| int | SupplyNoTerritories () |
| void | Tick (void) |
| void | UpdateQuality () |
|  | ~SkTerritories () |

|  |  |
| --- | --- |
| Public Attributes | |
| double | PolyHeightData [2500] |
| int | PolyRefData [2500] |
| int | PolySizeData [2500] |
| TTypesOfLandscapeElement | PolyTypeData [2500] |
| Landscape \* | TheLandscape |

|  |  |
| --- | --- |
| Private Member Functions | |
| void | DumpMapGraphics (const char \*a\_filename, Landscape \*a\_map) |
| int | EvaluateHabitatSquare (int xmin, int xmax, int ymin, int ymax, int NoPolygons) |
| int | PreEvaluateHabitat (int a\_x, int a\_y, int a\_range\_x, int a\_range\_y) |
| int | PreEvaluateHabitatStripX (int a\_x, int a\_y, int a\_range\_x) |
| int | PreEvaluateHabitatStripY (int a\_x, int a\_y, int a\_range\_x) |
| void | PreEvaluateQualGrid (SkQualGrid \*a\_grid, int a\_x, int a\_y, int a\_width, int a\_height) |
| void | PreFillQualCache (void) |
| void | PreFillQualGrid (void) |
| void | PreFillTerrPolyLists (skTTerritory \*a\_terr) |
| int | PreMakeForIterator (int a\_min\_incl, int a\_max\_excl, int \*a\_iter, int a\_norm\_max\_excl) |

|  |  |
| --- | --- |
| Private Attributes | |
| bool | Grid [1001][1001] |
| int | m\_for\_iter\_x [1000] |
| int | m\_for\_iter\_y [1000] |
| int | m\_hash\_size |
| int \* | m\_poly\_seen |
| int \* | m\_poly\_size |
| double \* | m\_qual\_cache |
| bool | m\_qual\_cache\_filled |
| SkQualGrid \*\* | m\_qual\_grid |
| int | m\_qual\_grid\_signal |
| int | m\_sim\_h\_div\_10 |
| int | m\_sim\_w\_div\_10 |
| int | NoTerritories |
| int | SimH |
| int | SimW |
| skTTerritory \* | Territories [400000] |

---

## Constructor & Destructor Documentation

|  |  |  |  |  |  |
| --- | --- | --- | --- | --- | --- |
| SkTerritories::SkTerritories | ( | Landscape \* | *L* | ) |  |

References NoTerritories, SimH, SimW, Landscape::SupplySimAreaHeight(), Landscape::SupplySimAreaWidth(), and TheLandscape.

{

TheLandscape = L;

NoTerritories = 0; // must be set to zero otherwise will try to delete

// non-existant territories on first call of PreProcessLandscape2()

SimW = L->SupplySimAreaWidth();

SimH = L->SupplySimAreaHeight();

}

|  |  |  |  |  |
| --- | --- | --- | --- | --- |
| SkTerritories::~SkTerritories | ( |  | ) |  |

References m\_poly\_seen, m\_poly\_size, m\_qual\_cache, m\_qual\_grid, m\_sim\_h\_div\_10, m\_sim\_w\_div\_10, NoTerritories, and Territories.

{

for ( int i = 0; i < NoTerritories; i++ ) delete Territories[ i ];

free( m\_poly\_seen );

free( m\_poly\_size );

free( m\_qual\_cache );

for ( int i = 0; i < m\_sim\_w\_div\_10 \* m\_sim\_h\_div\_10; i++ ) {

delete m\_qual\_grid[ i ];

}

free( m\_qual\_grid );

}

---

## Member Function Documentation

|  |  |  |  |
| --- | --- | --- | --- |
| void SkTerritories::ClaimGrid | ( | int | *x*, |
|  |  | int | *y*, |
|  |  | int | *range* |
|  | ) |  |  |

References Grid, m\_sim\_h\_div\_10, and m\_sim\_w\_div\_10.

Referenced by PreProcessLandscape2().

{

int l\_max\_x = x + range;

if ( l\_max\_x > m\_sim\_w\_div\_10 )

l\_max\_x = m\_sim\_w\_div\_10;

int l\_max\_y = y + range;

if ( l\_max\_y > m\_sim\_h\_div\_10 )

l\_max\_y = m\_sim\_h\_div\_10;

for ( int i = x; i < l\_max\_x; i++ ) {

for ( int j = y; j < l\_max\_y; j++ ) {

Grid[ i ] [ j ] = true;

}

}

}

|  |  |  |  |
| --- | --- | --- | --- |
| void SkTerritories::DumpMapGraphics | ( | const char \* | *a\_filename*, |
|  |  | Landscape \* | *a\_map* |
|  | ) |  |  |

private

References g\_msg, skTTerritory::m\_Location\_x, skTTerritory::m\_Location\_y, MapDumpColours, NoTerritories, skTTerritory::Size, Landscape::SupplyElementType(), Landscape::SupplySimAreaHeight(), Landscape::SupplySimAreaWidth(), Landscape::SupplyVegHeight(), Territories, tole\_Field, MapErrorMsg::Warn(), and WARN\_FILE.

{

int l\_width = a\_map->SupplySimAreaWidth();

int l\_height = a\_map->SupplySimAreaHeight();

unsigned int linesize = l\_width \* 3;

unsigned char \* frame\_buffer = ( unsigned char \* ) malloc( sizeof( unsigned char ) \* linesize \* l\_height );

FILE \* l\_file;

if ( frame\_buffer == NULL ) {

g\_msg->Warn( WARN\_FILE, "SkTerritories::DumpMapGraphics(): Out of memory!", "" );

exit( 1 );

}

for ( int y = 0, i = 0; y < l\_height; y++ ) {

for ( int x = 0; x < l\_width; x++ ) {

int eletype = ( int )a\_map->SupplyElementType( x, y );

int localcolor = MapDumpColours[ eletype ];

if ( eletype == ( int )tole\_Field ) {

int category;

double hei = a\_map->SupplyVegHeight( x, y );

if ( hei > 50.0 ) category = 0; else

category = ( int )( 200.0 - ( hei \* 4.0 ) );

localcolor = ( ( category \* 65536 ) + 65535 );

}

frame\_buffer[ i++ ] = (unsigned char)(localcolor & 0xff);

frame\_buffer[ i++ ] = (unsigned char)(( localcolor >> 8 ) & 0xff);

frame\_buffer[ i++ ] = (unsigned char) (( localcolor >> 16 ) & 0xff);

}

}

for ( int i = 0; i < NoTerritories; i++ ) {

int l\_x = Territories[ i ]->m\_Location\_x;

int l\_y = Territories[ i ]->m\_Location\_y;

int l\_s = Territories[ i ]->Size;

// Horizontal lines of territory bounding box.

for ( int x = l\_x; x < l\_x + l\_s; x++ ) {

int y = l\_y;

if ( x >= 0 && x < l\_width && y >= 0 && y < l\_height ) {

int pixel\_p = x \* 3 + linesize \* y;

frame\_buffer[ pixel\_p++ ] = 200;

frame\_buffer[ pixel\_p++ ] = 0;

frame\_buffer[ pixel\_p ] = 0;

}

y = l\_y + l\_s - 1;

if ( x >= 0 && x < l\_width && y >= 0 && y < l\_height ) {

int pixel\_p = x \* 3 + linesize \* y;

frame\_buffer[ pixel\_p++ ] = 200;

frame\_buffer[ pixel\_p++ ] = 0;

frame\_buffer[ pixel\_p ] = 0;

}

}

for ( int y = l\_y; y < l\_y + l\_s; y++ ) {

int x = l\_x;

if ( x >= 0 && x < l\_width && y >= 0 && y < l\_height ) {

int pixel\_p = x \* 3 + linesize \* y;

frame\_buffer[ pixel\_p++ ] = 200;

frame\_buffer[ pixel\_p++ ] = 0;

frame\_buffer[ pixel\_p ] = 0;

}

x = l\_x + l\_s - 1;

if ( x >= 0 && x < l\_width && y >= 0 && y < l\_height ) {

int pixel\_p = x \* 3 + linesize \* y;

frame\_buffer[ pixel\_p++ ] = 200;

frame\_buffer[ pixel\_p++ ] = 0;

frame\_buffer[ pixel\_p ] = 0;

}

}

}

l\_file = fopen(a\_filename, "w" );

if ( !l\_file ) {

g\_msg->Warn( WARN\_FILE, "SkTerritories::DumpMapGraphics(): ""Unable to open file for writing: %s\n", a\_filename );

exit( 1 );

}

fprintf( l\_file, "P6\n%d %d %d\n", l\_width, l\_height, 255 );

fwrite( frame\_buffer, sizeof( unsigned char ), linesize \* l\_height, l\_file );

fclose( l\_file );

free( frame\_buffer );

}

|  |  |  |  |  |  |
| --- | --- | --- | --- | --- | --- |
| void SkTerritories::EvaluateAllTerritories | ( | void |  | ) |  |

References EvaluateHabitatN(), NoTerritories, and Territories.

Referenced by Skylark\_Population\_Manager::DoFirst().

{

for ( int i = 0; i < NoTerritories; i++ ) {

EvaluateHabitatN(Territories[i]);

}

}

|  |  |  |  |  |  |
| --- | --- | --- | --- | --- | --- |
| double SkTerritories::EvaluateHabitatN | ( | skTTerritory \* | *a\_terr* | ) |  |

References skTTerritory::m\_competitionscaler, skTTerritory::m\_polys, skTTerritory::m\_sizes, PrePolyNQual(), skTTerritory::SetQuality(), and Landscape::Warn().

Referenced by EvaluateAllTerritories().

{

//a\_good\_polys = 0;

int a\_area\_polys = 0;

int l\_polygons = (int) a\_terr->m\_polys.size();

double l\_qual = 0.0;

if ( l\_polygons > 2500 ) {

g\_land->Warn( "SkTerritories::EvaluateHabitat1(): - Polygons > 2500", "" );

exit( 1 );

}

for ( int i = 0; i < l\_polygons; i++ ) {

int l\_poly = a\_terr->m\_polys[ i ];

l\_qual += ( double )( a\_terr->m\_sizes[ i ] ) \* PrePolyNQual( l\_poly, 0 );

//l\_qual += ( double )( a\_terr->m\_sizes[ i ] ) \* PrePoly2Qual( l\_poly );

a\_area\_polys += a\_terr->m\_sizes[ i ];

}

l\_qual \*= ( a\_terr->m\_competitionscaler); //\* a\_terr->GetHeterogeneity());

// Update territory quality

a\_terr->SetQuality((int) l\_qual);

// return the quality to calling method

return l\_qual;

}

|  |  |  |  |
| --- | --- | --- | --- |
| int SkTerritories::EvaluateHabitatSquare | ( | int | *xmin*, |
|  |  | int | *xmax*, |
|  |  | int | *ymin*, |
|  |  | int | *ymax*, |
|  |  | int | *NoPolygons* |
|  | ) |  |  |

private

|  |  |  |  |
| --- | --- | --- | --- |
| void SkTerritories::FemaleOccupy | ( | int | *ref*, |
|  |  | Skylark\_Female \* | *Female* |
|  | ) |  |  |

References skTTerritory::F\_Owner, and Territories.

Referenced by Skylark\_Female::EstablishTerritory(), and Skylark\_Male::OnReHouse().

{

Territories[ ref ]->F\_Owner = Female;

}

|  |  |  |  |
| --- | --- | --- | --- |
| void SkTerritories::GetTerritoriesByDistance | ( | int | *nx*, |
|  |  | int | *ny*, |
|  |  | vector< Point > \* | *alist* |
|  | ) |  |  |

References CompareDist(), Point::m\_x, Point::m\_y, NoTerritories, and Territories.

Referenced by Skylark\_Male::OnEvicted(), and Skylark\_Male::st\_Arriving().

{

int dist=0;

// alist->resize(NoTerritories);

Point p;

for ( int i = 0; i < NoTerritories; i++ ) {

dist = abs(Territories[ i ]->m\_Location\_x-nx);

dist += abs(Territories[ i ]->m\_Location\_y-ny);

p.m\_x = i;

p.m\_y = dist;

alist->push\_back(p);

}

sort(alist->begin(), alist->end(), CompareDist);

}

|  |  |  |  |
| --- | --- | --- | --- |
| bool SkTerritories::IsExtGridPositionValid | ( | int & | *x*, |
|  |  | int & | *y*, |
|  |  | int | *range* |
|  | ) |  |  |

References Grid, m\_sim\_h\_div\_10, and m\_sim\_w\_div\_10.

Referenced by PreProcessLandscape2().

{

// The commented out code \*\*CJT\*\* below could be used if we want

// to invalidate the whole area up to the point where we hit problems

// However, this will lead to large territory-less holes in the

// landscape

int l\_max\_x = x + range;

if ( l\_max\_x > m\_sim\_w\_div\_10 ) {

// l\_max\_x = m\_sim\_w\_div\_10;

return false;

}

int l\_max\_y = y + range;

if ( l\_max\_y > m\_sim\_h\_div\_10 ) {

//l\_max\_y = m\_sim\_h\_div\_10;

return false;

}

//Needs only to check the left most column and bottom row

// The leftmost column

for ( int j = y; j < l\_max\_y; j++ ) {

if ( Grid[ x ] [ j ] ) {

return false;

}

}

// The bottom row

for ( int i = x; i < l\_max\_x; i++ ) {

if ( Grid[ i ] [ y ] ) {

return false;

}

}

// All OK

return true;

}

|  |  |  |  |
| --- | --- | --- | --- |
| bool SkTerritories::IsGridPositionValid | ( | int & | *x*, |
|  |  | int & | *y*, |
|  |  | int | *range* |
|  | ) |  |  |

References Grid, m\_sim\_h\_div\_10, and m\_sim\_w\_div\_10.

Referenced by PreProcessLandscape2().

{

int l\_max\_x = x + range;

if ( l\_max\_x > m\_sim\_w\_div\_10 ) {

//l\_max\_x = m\_sim\_w\_div\_10;

return false;

}

int l\_max\_y = y + range;

if ( l\_max\_y > m\_sim\_h\_div\_10 ) {

//l\_max\_y = m\_sim\_h\_div\_10;

return false;

}

for ( int i = x; i < l\_max\_x; i++ ) {

for ( int j = y; j < l\_max\_y; j++ ) {

if ( Grid[ i ] [ j ] ) {

return false;

}

}

}

// No occupied squares found so

return true;

}

|  |  |  |  |
| --- | --- | --- | --- |
| int SkTerritories::IsValid | ( | int | *nx*, |
|  |  | int | *ny* |
|  | ) |  |  |

References NoTerritories, and Territories.

{

int found = -1;

for ( int i = 0; i < NoTerritories; i++ ) {

if ( ( Territories[ i ]->m\_Location\_x <= nx ) && ( Territories[ i ]->m\_Location\_x + Territories[ i ]->Size > nx )

&& ( Territories[ i ]->m\_Location\_y <= ny ) && ( Territories[ i ]->m\_Location\_y + Territories[ i ]->Size > ny ) ) {

found = i;

break;

}

}

return found;

}

|  |  |  |  |
| --- | --- | --- | --- |
| void SkTerritories::Occupy | ( | int | *ref*, |
|  |  | Skylark\_Male \* | *Male* |
|  | ) |  |  |

References skTTerritory::Owner, and Territories.

Referenced by Skylark\_Male::EstablishingATerritory().

{

Territories[ ref ]->Owner = Male;

}

|  |  |  |  |  |  |
| --- | --- | --- | --- | --- | --- |
| void SkTerritories::PreCachePoly | ( | int | *a\_poly* | ) |  |

References m\_qual\_cache, and PrePoly2Qual().

Referenced by Landscape::SkylarkEvaluation().

{

m\_qual\_cache[a\_poly] = PrePoly2Qual(a\_poly);

}

|  |  |  |  |
| --- | --- | --- | --- |
| int SkTerritories::PreEvaluateHabitat | ( | int | *a\_x*, |
|  |  | int | *a\_y*, |
|  |  | int | *a\_range\_x*, |
|  |  | int | *a\_range\_y* |
|  | ) |  |  |

inlineprivate

References SkQualGrid::m\_qual, m\_qual\_grid, m\_sim\_h\_div\_10, and m\_sim\_w\_div\_10.

Referenced by PreProcessLandscape2().

{

double l\_qual = 0.0;

int l\_max\_x = a\_x + a\_range\_x;

int l\_max\_y = a\_y + a\_range\_y;

if ( l\_max\_x > m\_sim\_w\_div\_10 )

l\_max\_x = m\_sim\_w\_div\_10;

if ( l\_max\_y > m\_sim\_h\_div\_10 )

l\_max\_y = m\_sim\_h\_div\_10;

for ( int x = a\_x; x < l\_max\_x; x++ ) {

for ( int y = a\_y; y < l\_max\_y; y++ ) {

int l\_i = x + y \* m\_sim\_w\_div\_10;

l\_qual += m\_qual\_grid[ l\_i ]->m\_qual;

}

}

return ( int )l\_qual;

}

|  |  |  |  |
| --- | --- | --- | --- |
| int SkTerritories::PreEvaluateHabitatStripX | ( | int | *a\_x*, |
|  |  | int | *a\_y*, |
|  |  | int | *a\_range\_x* |
|  | ) |  |  |

inlineprivate

References SkQualGrid::m\_qual, m\_qual\_grid, and m\_sim\_w\_div\_10.

Referenced by PreProcessLandscape2().

{

double l\_qual = 0.0;

int l\_y\_fixed = a\_y \* m\_sim\_w\_div\_10;

int l\_max\_x = a\_x + a\_range\_x;

if ( l\_max\_x > m\_sim\_w\_div\_10 )

l\_max\_x = m\_sim\_w\_div\_10;

for ( int x = a\_x; x < l\_max\_x; x++ ) {

int l\_i = x + l\_y\_fixed;

l\_qual+=m\_qual\_grid[ l\_i ]->m\_qual;

/\*

int l\_gmax = (int)m\_qual\_grid[ l\_i ]->m\_polys.size();

for ( int g = 0; g < l\_gmax; g++ ) {

l\_qual += ( double )( m\_qual\_grid[ l\_i ]->m\_sizes[ g ] ) \* m\_qual\_cache[ m\_qual\_grid[ l\_i ]->m\_polys[ g ]];

}

\*/

}

return ( int )l\_qual;

}

|  |  |  |  |
| --- | --- | --- | --- |
| int SkTerritories::PreEvaluateHabitatStripY | ( | int | *a\_x*, |
|  |  | int | *a\_y*, |
|  |  | int | *a\_range\_x* |
|  | ) |  |  |

inlineprivate

References SkQualGrid::m\_qual, m\_qual\_grid, m\_sim\_h\_div\_10, and m\_sim\_w\_div\_10.

Referenced by PreProcessLandscape2().

{

double l\_qual = 0.0;

int l\_max\_y = a\_y + a\_range\_y;

if ( l\_max\_y > m\_sim\_h\_div\_10 ) l\_max\_y = m\_sim\_h\_div\_10;

for ( int y = a\_y; y < l\_max\_y; y++ ) {

int l\_i = a\_x + y \* m\_sim\_w\_div\_10;

l\_qual += m\_qual\_grid[ l\_i ]->m\_qual;

/\*

int l\_gmax = (int)m\_qual\_grid[ l\_i ]->m\_polys.size();

for ( int g = 0; g < l\_gmax; g++ ) {

l\_qual += ( double )( m\_qual\_grid[ l\_i ]->m\_sizes[ g ] ) \* m\_qual\_cache[ m\_qual\_grid[ l\_i ]->m\_polys[ g ]];

}

\*/

}

return ( int )l\_qual;

}

|  |  |  |  |
| --- | --- | --- | --- |
| void SkTerritories::PreEvaluateQualGrid | ( | SkQualGrid \* | *a\_grid*, |
|  |  | int | *a\_x*, |
|  |  | int | *a\_y*, |
|  |  | int | *a\_width*, |
|  |  | int | *a\_height* |
|  | ) |  |  |

private

References SkQualGrid::Insert(), m\_poly\_seen, SkQualGrid::m\_polys, SkQualGrid::m\_qual, m\_qual\_cache, SkQualGrid::m\_sizes, PolyRefData, Landscape::SupplyPolyRef(), and TheLandscape.

Referenced by PreFillQualGrid().

{

int l\_polygons = 0;

double qual=0;

if (a\_grid->m\_polys.size()==0) {

for ( int y = a\_y; y < a\_y + a\_height; y++ ) {

for ( int x = a\_x; x < a\_x + a\_width; x++ ) {

int l\_poly = TheLandscape->SupplyPolyRef( x, y );

int l\_i = m\_poly\_seen[ l\_poly ];

if ( l\_i >= 0 ) {

a\_grid->m\_sizes[ l\_i ] ++;

continue;

}

m\_poly\_seen[ l\_poly ] = a\_grid->Insert( l\_poly );

PolyRefData[ l\_polygons++ ] = l\_poly;

}

}

for ( int i = 0; i < (int) a\_grid->m\_polys.size(); i++ ) {

qual += ( a\_grid->m\_sizes[ i ] ) \* m\_qual\_cache[ a\_grid->m\_polys[ i ]];

m\_poly\_seen[ PolyRefData[ i ]] = -1; // Get ready for the next square - done here because here we know how may -1s to use

}

}

else

{

for ( int i = 0; i < (int) a\_grid->m\_polys.size(); i++ ) {

qual += ( a\_grid->m\_sizes[ i ] ) \* m\_qual\_cache[ a\_grid->m\_polys[ i ]];

}

}

a\_grid->m\_qual=qual;

}

|  |  |  |  |  |  |
| --- | --- | --- | --- | --- | --- |
| void SkTerritories::PreFillQualCache | ( | void |  | ) |  |

private

This method is called only once since the male cues on returning to the landscape are thought to be structural and not vegatation based (e.g. he waits in ploughed fields). Later he will use the same assessment as the females, but this is not possible early in the season before vegetation grows.

References m\_hash\_size, m\_poly\_seen, SkQualGrid::m\_polys, m\_qual\_cache, m\_qual\_cache\_filled, m\_qual\_grid, m\_sim\_h\_div\_10, m\_sim\_w\_div\_10, and PrePoly2Qual().

{

int l\_dummy = 0;

m\_qual\_cache\_filled = true;

for ( int i = 0; i < m\_sim\_w\_div\_10 \* m\_sim\_h\_div\_10; i++ ) {

int l\_gmax = (int)m\_qual\_grid[ i ]->m\_polys.size();

for ( int g = 0; g < l\_gmax; g++ ) {

int l\_this\_poly = m\_qual\_grid[ i ]->m\_polys[ g ];

if ( m\_poly\_seen[ l\_this\_poly ] == -1 ) {

m\_poly\_seen[ l\_this\_poly ] = 1;

m\_qual\_cache[ l\_this\_poly ] = PrePoly2Qual( l\_this\_poly );

//m\_qual\_cache[ l\_this\_poly ] = PrePolyNQual( l\_this\_poly, & l\_dummy );

}

}

}

for ( int i = 0; i < m\_hash\_size; i++ ) {

m\_poly\_seen[ i ] = -1; // m\_poly\_seen is used elsewhere so is reset here.

}

}

|  |  |  |  |  |  |
| --- | --- | --- | --- | --- | --- |
| void SkTerritories::PreFillQualGrid | ( | void |  | ) |  |

private

References g\_msg, m\_hash\_size, m\_poly\_seen, m\_poly\_size, m\_qual\_cache, m\_qual\_grid, m\_qual\_grid\_signal, m\_sim\_h\_div\_10, m\_sim\_w\_div\_10, PreEvaluateQualGrid(), SimH, SimW, Landscape::SkylarkEvaluation(), Landscape::SupplyLargestPolyNumUsed(), TheLandscape, MapErrorMsg::Warn(), and WARN\_FILE.

Referenced by PreProcessLandscape2().

{

// if ( m\_qual\_grid\_signal == 2 )

// return;

if ( m\_qual\_grid\_signal != 2 ){

// First time we are ever called. Allocate the memory for the

// quality computations, and fill in the 10x10m quality grid.

m\_qual\_grid\_signal = 2;

m\_hash\_size = g\_land->SupplyLargestPolyNumUsed() + 1;

m\_sim\_w\_div\_10 = SimW / 10;

m\_sim\_h\_div\_10 = SimH / 10;

// Using malloc because this allows us to use a test of available memory

m\_qual\_grid = ( SkQualGrid \* \* ) malloc( sizeof( SkQualGrid \* ) \* m\_sim\_w\_div\_10 \* m\_sim\_h\_div\_10 );

m\_poly\_seen = ( int \* ) malloc( sizeof( int ) \* m\_hash\_size );

m\_poly\_size = ( int \* ) malloc( sizeof( int ) \* m\_hash\_size );

m\_qual\_cache = ( double \* ) malloc( sizeof( double ) \* m\_hash\_size );

bool l\_ok = true;

for ( int i = 0; i < m\_sim\_w\_div\_10 \* m\_sim\_h\_div\_10; i++ ) {

m\_qual\_grid[ i ] = new SkQualGrid;

if ( m\_qual\_grid[ i ] == NULL ) {

l\_ok = false;

break;

}

}

if ( m\_qual\_grid == NULL || m\_poly\_seen == NULL || m\_qual\_cache == NULL || m\_poly\_size == NULL || !l\_ok ) {

g\_msg->Warn( WARN\_FILE, "SkTerritories::PreFillQualGrid(): ""Out of memory!\n", "" );

exit( 1 );

}

}

for ( int i = 0; i < m\_hash\_size; i++ ) {

m\_poly\_seen[ i ] = -1;

}

TheLandscape->SkylarkEvaluation( this );

int i = 0;

for ( int y = 0; y < m\_sim\_h\_div\_10; y++ ) {

for ( int x = 0; x < m\_sim\_w\_div\_10; x++ ) {

PreEvaluateQualGrid( m\_qual\_grid[ i++ ], x \* 10, y \* 10, 10, 10 );

}

}

//PreFillQualCache();

}

|  |  |  |  |  |  |
| --- | --- | --- | --- | --- | --- |
| void SkTerritories::PreFillTerrPolyLists | ( | skTTerritory \* | *a\_terr* | ) |  |

private

References HomeRangeDiameterDiv20, m\_for\_iter\_x, m\_for\_iter\_y, skTTerritory::m\_hr\_polys, skTTerritory::m\_hr\_sizes, m\_poly\_seen, m\_poly\_size, skTTerritory::m\_polys, SkQualGrid::m\_polys, m\_qual\_grid, skTTerritory::m\_range\_div10, m\_sim\_h\_div\_10, m\_sim\_w\_div\_10, skTTerritory::m\_sizes, SkQualGrid::m\_sizes, skTTerritory::m\_x\_div10, skTTerritory::m\_y\_div10, PolyRefData, and PreMakeForIterator().

Referenced by PreProcessLandscape2(), and Split().

{

int x\_min\_inc = a\_terr->m\_x\_div10;

int x\_max\_exc = a\_terr->m\_x\_div10 + a\_terr->m\_range\_div10;

int y\_min\_inc = a\_terr->m\_y\_div10;

int y\_max\_exc = a\_terr->m\_y\_div10 + a\_terr->m\_range\_div10;

int l\_polygons = 0;

for ( int y = y\_min\_inc; y < y\_max\_exc; y++ ) {

for ( int x = x\_min\_inc; x < x\_max\_exc; x++ ) {

int l\_i = x + y \* m\_sim\_w\_div\_10;

int l\_gmax = (int)m\_qual\_grid[ l\_i ]->m\_polys.size();

for ( int g = 0; g < l\_gmax; g++ ) {

int l\_poly = m\_qual\_grid[ l\_i ]->m\_polys[ g ];

if ( m\_poly\_seen[ l\_poly ] == 1 ) {

m\_poly\_size[ l\_poly ] += m\_qual\_grid[ l\_i ]->m\_sizes[ g ];

continue;

}

m\_poly\_seen[ l\_poly ] = 1;

m\_poly\_size[ l\_poly ] = m\_qual\_grid[ l\_i ]->m\_sizes[ g ];

PolyRefData[ l\_polygons++ ] = l\_poly;

}

}

}

a\_terr->m\_polys.resize( l\_polygons );

a\_terr->m\_sizes.resize( l\_polygons );

for ( int i = 0; i < l\_polygons; i++ ) {

int l\_poly = PolyRefData[ i ];

m\_poly\_seen[ l\_poly ] = -1;

a\_terr->m\_polys[ i ] = l\_poly;

a\_terr->m\_sizes[ i ] = m\_poly\_size[ l\_poly ];

}

// Now build the same lists for the home range,

// centered on this territory.

int l\_offset = ( a\_terr->m\_range\_div10 ) >> 1;

x\_min\_inc = a\_terr->m\_x\_div10 + l\_offset - HomeRangeDiameterDiv20;

x\_max\_exc = a\_terr->m\_x\_div10 + l\_offset + HomeRangeDiameterDiv20;

y\_min\_inc = a\_terr->m\_y\_div10 + l\_offset - HomeRangeDiameterDiv20;

y\_max\_exc = a\_terr->m\_y\_div10 + l\_offset + HomeRangeDiameterDiv20;

l\_polygons = 0;

int l\_c = PreMakeForIterator( x\_min\_inc, x\_max\_exc, m\_for\_iter\_x, m\_sim\_w\_div\_10 );

PreMakeForIterator( y\_min\_inc, y\_max\_exc, m\_for\_iter\_y, m\_sim\_h\_div\_10 );

for ( int l\_y = 0; l\_y < l\_c; l\_y++ ) {

int y = m\_for\_iter\_y[ l\_y ];

for ( int l\_x = 0; l\_x < l\_c; l\_x++ ) {

int l\_i = m\_for\_iter\_x[ l\_x ] + y \* m\_sim\_w\_div\_10;

int l\_gmax = (int)m\_qual\_grid[ l\_i ]->m\_polys.size();

for ( int g = 0; g < l\_gmax; g++ ) {

int l\_poly = m\_qual\_grid[ l\_i ]->m\_polys[ g ];

if ( m\_poly\_seen[ l\_poly ] == 1 ) {

m\_poly\_size[ l\_poly ] += m\_qual\_grid[ l\_i ]->m\_sizes[ g ];

continue;

}

m\_poly\_seen[ l\_poly ] = 1;

m\_poly\_size[ l\_poly ] = m\_qual\_grid[ l\_i ]->m\_sizes[ g ];

PolyRefData[ l\_polygons++ ] = l\_poly;

}

}

}

a\_terr->m\_hr\_polys.resize( l\_polygons );

a\_terr->m\_hr\_sizes.resize( l\_polygons );

for ( int i = 0; i < l\_polygons; i++ ) {

int l\_poly = PolyRefData[ i ];

m\_poly\_seen[ l\_poly ] = -1;

a\_terr->m\_hr\_polys[ i ] = l\_poly;

a\_terr->m\_hr\_sizes[ i ] = m\_poly\_size[ l\_poly ];

}

}

|  |  |  |  |
| --- | --- | --- | --- |
| int SkTerritories::PreMakeForIterator | ( | int | *a\_min\_incl*, |
|  |  | int | *a\_max\_excl*, |
|  |  | int \* | *a\_iter*, |
|  |  | int | *a\_norm\_max\_excl* |
|  | ) |  |  |

private

Referenced by PreFillTerrPolyLists().

{

int i, index = 0;

int med\_max;

int high\_max = -1;

if ( a\_max\_excl >= a\_norm\_max\_excl ) {

med\_max = a\_norm\_max\_excl;

high\_max = a\_max\_excl;

} else {

med\_max = a\_max\_excl;

}

for ( i = a\_min\_incl; i < 0; i++ ) {

a\_iter[ index++ ] = i + a\_norm\_max\_excl;

}

for ( ; i < med\_max; i++ ) {

a\_iter[ index++ ] = i;

}

for ( ; i < high\_max; i++ ) {

a\_iter[ index++ ] = i - a\_norm\_max\_excl;

}

return index;

}

|  |  |  |  |  |  |
| --- | --- | --- | --- | --- | --- |
| double SkTerritories::PrePoly2Qual | ( | int | *a\_poly* | ) |  |

inline

Used only on start-up when potential territories need to be determined.

References HQualityBareEarth, HQualityHedge, HQualityHedgeScrub, HQualityMetalRoad, HQualityTall, HQualityTall2, HQualityTallVeg, HQualityWater, PatchyPremium, SKOPTIMALHABITATSCORE, Landscape::SupplyElementType(), Landscape::SupplyVegHeight(), Landscape::SupplyVegPatchy(), TheLandscape, tole\_ActivePit, tole\_BeetleBank, tole\_Building, tole\_Coast, tole\_ConiferousForest, tole\_DeciduousForest, tole\_Field, tole\_FieldBoundary, tole\_Foobar, tole\_Freshwater, tole\_Garden, tole\_Heath, tole\_HedgeBank, tole\_Hedges, tole\_LargeRoad, tole\_Marsh, tole\_MixedForest, tole\_NaturalGrass, tole\_PermanentSetaside, tole\_PermPasture, tole\_PermPastureLowYield, tole\_PermPastureTussocky, tole\_PitDisused, tole\_Railway, tole\_River, tole\_RiversidePlants, tole\_RiversideTrees, tole\_RoadsideVerge, tole\_Saltwater, tole\_Scrub, tole\_SmallRoad, tole\_StoneWall, tole\_Track, tole\_UnsprayedFieldMargin, tole\_Urban, tole\_YoungForest, and Landscape::Warn().

Referenced by PreCachePoly(), and PreFillQualCache().

{

TTypesOfLandscapeElement l\_type = TheLandscape->SupplyElementType( a\_poly );

double l\_height;

double l\_score = -9999.0;

// bool ppremium = false;

// int l\_index;

switch ( l\_type ) {

case tole\_YoungForest: //

l\_score = HQualityBareEarth + PatchyPremium;

break;

case tole\_Hedges: // 130

case tole\_HedgeBank:

l\_score = HQualityHedge;

break;

case tole\_PitDisused: // 75

case tole\_RiversideTrees: // 97

case tole\_DeciduousForest: // 40

case tole\_MixedForest: // 60

case tole\_ConiferousForest: // 50

case tole\_ActivePit: // 115

case tole\_Building: // 5

l\_score = HQualityTall2;

break;

case tole\_Garden: //11

case tole\_Urban: // 10

l\_score = HQualityTallVeg;

break;

case tole\_Marsh: // 95

case tole\_Scrub: // 70

l\_score = HQualityHedgeScrub;

break;

case tole\_Field:

case tole\_RoadsideVerge: // 13

case tole\_FieldBoundary: // 160

case tole\_UnsprayedFieldMargin:

case tole\_PermanentSetaside: // 7

case tole\_Heath:

case tole\_PermPastureLowYield:

case tole\_PermPastureTussocky:

case tole\_PermPasture: // 35

case tole\_NaturalGrass: // 110

case tole\_BeetleBank:

// Must be potentially suitable vegetation element so score depends on

// the vegetation height

l\_height = TheLandscape->SupplyVegHeight( a\_poly );

if ( l\_height >= 200.0 )

{

l\_score = HQualityTallVeg;

} else

{

if ( l\_height >= 110.0 )

{

l\_score = HQualityTall;

} else

{

l\_score = SKOPTIMALHABITATSCORE; // Start with a max

if (!TheLandscape->SupplyVegPatchy( a\_poly )) l\_score \*= 0.50;

}

}

break;

case tole\_SmallRoad: // 120

case tole\_LargeRoad: // 121

l\_score = HQualityMetalRoad;

break;

case tole\_Freshwater: // 90

case tole\_River: // 96

case tole\_Saltwater: // 80

case tole\_Coast: // 100

case tole\_RiversidePlants: // 98

case tole\_StoneWall: // 15

case tole\_Track: // 123

case tole\_Railway: // 118

l\_score = HQualityWater; // Neutral

break;

case tole\_Foobar: // 999 !! type unknown - should not happen

default:

static char l\_errornum[ 20 ];

sprintf( l\_errornum, "%d", l\_type );

TheLandscape->Warn( "SkTerritories::PrePoly2Qual(): ""Unknown tole\_type", l\_errornum );

exit( 1 );

break;

}

if (TheLandscape->SupplyVegPatchy( a\_poly )) l\_score += PatchyPremium;

if (l\_score > SKOPTIMALHABITATSCORE) l\_score = SKOPTIMALHABITATSCORE;

return l\_score;

}

|  |  |  |  |
| --- | --- | --- | --- |
| double SkTerritories::PrePolyNQual | ( | int | *a\_poly*, |
|  |  | int \* | *a\_good\_polys* |
|  | ) |  |  |

inline

References DensityScore, HeightScore, HQualityBareEarth, HQualityHedge, HQualityHedgeScrub, HQualityMetalRoad, HQualityTall, HQualityTall2, HQualityTallVeg, HQualityWater, PatchyPremium, SKOPTIMALHABITATSCORE, Landscape::SupplyElementType(), Landscape::SupplyHasTramlines(), Landscape::SupplySkScrapes(), Landscape::SupplyVegDensity(), Landscape::SupplyVegHeight(), Landscape::SupplyVegPatchy(), TheLandscape, tole\_ActivePit, tole\_BeetleBank, tole\_Building, tole\_Coast, tole\_ConiferousForest, tole\_DeciduousForest, tole\_Field, tole\_FieldBoundary, tole\_Foobar, tole\_Freshwater, tole\_Garden, tole\_Heath, tole\_HedgeBank, tole\_Hedges, tole\_LargeRoad, tole\_Marsh, tole\_MixedForest, tole\_NaturalGrass, tole\_PermanentSetaside, tole\_PermPasture, tole\_PermPastureLowYield, tole\_PermPastureTussocky, tole\_PitDisused, tole\_Railway, tole\_River, tole\_RiversidePlants, tole\_RiversideTrees, tole\_RoadsideVerge, tole\_Saltwater, tole\_Scrub, tole\_SmallRoad, tole\_StoneWall, tole\_Track, tole\_UnsprayedFieldMargin, tole\_Urban, tole\_YoungForest, CfgInt::value(), CfgFloat::value(), and Landscape::Warn().

Referenced by EvaluateHabitatN().

{

TTypesOfLandscapeElement l\_type = TheLandscape->SupplyElementType( a\_poly );

double l\_height;

double l\_score = -9999.0;

bool ppremium = false;

// int l\_index;

switch ( l\_type ) {

case tole\_YoungForest: //

l\_score = HQualityBareEarth + PatchyPremium;

break;

case tole\_Hedges: // 130

case tole\_HedgeBank:

l\_score = HQualityHedge;

break;

case tole\_PitDisused: // 75

case tole\_RiversideTrees: // 97

case tole\_DeciduousForest: // 40

case tole\_MixedForest: // 60

case tole\_ConiferousForest: // 50

case tole\_ActivePit: // 115

case tole\_Building: // 5

l\_score = HQualityTall2;

break;

case tole\_Garden: //11

case tole\_Urban: // 10

l\_score = HQualityTallVeg;

break;

case tole\_Marsh: // 95

case tole\_Scrub: // 70

l\_score = HQualityHedgeScrub;

break;

case tole\_Field:

case tole\_RoadsideVerge: // 13

case tole\_FieldBoundary: // 160

case tole\_UnsprayedFieldMargin:

case tole\_PermanentSetaside: // 7

case tole\_Heath:

case tole\_PermPastureLowYield:

case tole\_PermPastureTussocky:

case tole\_PermPasture: // 35

case tole\_NaturalGrass: // 110

case tole\_BeetleBank:

// Must be potentially suitable vegetation element so score depends on

// the vegetation height

l\_height = TheLandscape->SupplyVegHeight( a\_poly );

ppremium = TheLandscape->SupplyVegPatchy( a\_poly );

// If using skylark scrapes then we need the code below

if ( TheLandscape->SupplySkScrapes(a\_poly) && (l\_height >=30) )

{

if ( l\_height >= 200.0 )

{

l\_score = HQualityTallVeg;

} else

{

if ( l\_height >= 110.0 )

{

l\_score = HQualityTall;

}

else if ( l\_height < 3.0 )

{

l\_score = HQualityBareEarth;

} else

{

l\_score = SKOPTIMALHABITATSCORE / 2.0; // Start with standard (less some to allow for doubling territory)

double hh;

if (l\_height>70) hh = 70; else hh = l\_height;

l\_score += hh \* cfg\_SkScrapesPremiumII.value();

int density = TheLandscape->SupplyVegDensity( a\_poly );

l\_score += ( DensityScore[ density ] + HeightScore[ ( int )l\_height ] );

}

}

}

else

{

// No skylark scrapes

if ( l\_height >= 200.0 )

{

l\_score = HQualityTallVeg;

} else

{

if ( l\_height >= 110.0 )

{

l\_score = HQualityTall;

if ( ppremium ) l\_score += PatchyPremium;

}

else if ( l\_height < 3.0 )

{

l\_score = HQualityBareEarth;

} else

{

l\_score = SKOPTIMALHABITATSCORE / 2.0; // Start with half standard - less some to allow doubling (taken up in the minimum territory accept score)

int density;

if (l\_height<=30) density = 0; else density = TheLandscape->SupplyVegDensity( a\_poly );

l\_score += ( DensityScore[ density ] + HeightScore[ ( int )l\_height ] );

if ( ppremium ) l\_score += PatchyPremium;

else

{

if ( TheLandscape->SupplyHasTramlines( a\_poly )) l\_score += cfg\_SkTramlinesPremium.value();

}

}

}

}

break;

case tole\_SmallRoad: // 120

case tole\_LargeRoad: // 121

l\_score = HQualityMetalRoad;

break;

case tole\_Freshwater: // 90

case tole\_River: // 96

case tole\_Saltwater: // 80

case tole\_Coast: // 100

case tole\_RiversidePlants: // 98

case tole\_StoneWall: // 15

case tole\_Track: // 123

case tole\_Railway: // 118

l\_score = HQualityWater; // Neutral

break;

case tole\_Foobar: // 999 !! type unknown - should not happen

default:

static char l\_errornum[ 20 ];

sprintf( l\_errornum, "%d", l\_type );

TheLandscape->Warn( "SkTerritories::PrePoly2Qual(): ""Unknown tole\_type", l\_errornum );

exit( 1 );

break;

}

if (l\_score > SKOPTIMALHABITATSCORE) l\_score = SKOPTIMALHABITATSCORE;

return l\_score;

}

|  |  |  |  |  |  |
| --- | --- | --- | --- | --- | --- |
| void SkTerritories::PreProcessLandscape2 | ( | Landscape \* | *L* | ) |  |

References ClaimGrid(), g\_rand\_uni, Grid, IsExtGridPositionValid(), IsGridPositionValid(), m\_sim\_h\_div\_10, m\_sim\_w\_div\_10, NoTerritories, PreEvaluateHabitat(), PreEvaluateHabitatStripX(), PreEvaluateHabitatStripY(), PreFillQualGrid(), PreFillTerrPolyLists(), random(), Territories, skTTerritory::TestNestPossibility(), CfgFloat::value(), and Landscape::Warn().

Referenced by Skylark\_Population\_Manager::DoFirst().

{

//time\_t l\_time = time( NULL );

//printf("%s\n", asctime(localtime(&l\_time)));

// if this is not the first year then delete the old territories

for ( int i = 0; i < NoTerritories; i++ )

delete Territories[ i ];

NoTerritories = 0;

PreFillQualGrid();

// Zero all Grid Positions

for ( int i = 0; i < m\_sim\_w\_div\_10; i++ )

for ( int j = 0; j < m\_sim\_h\_div\_10; j++ )

Grid[ i ] [ j ] = false;

int x = random( m\_sim\_w\_div\_10 ); // was 3

int y = random( m\_sim\_h\_div\_10 ); // was 0

int qual;

int range = 5; // Start with a 50m territory

// Next start the loop

int tries = m\_sim\_w\_div\_10 \* m\_sim\_h\_div\_10;

while ( tries-- ) {

int targetqual = (int) (cfg\_FemaleMinTerritoryAcceptScore.value() \* (0.9 + g\_rand\_uni()/5.0));

if ( !IsGridPositionValid( x, y, range ) ) {

if ( ++x >= m\_sim\_w\_div\_10 ) {

x = random( 3 );

if ( ++y >= m\_sim\_h\_div\_10 ) {

y = 0;

}

}

continue;

}

// Location was OK, ie. not occupied.

// Try the smallest possible territory located right at our starting

// position.

qual = PreEvaluateHabitat( x, y, range, range );

// Is it suitable?

if ( qual >= targetqual ) {

// If so create it and claim the area on the grid

// Is OK so create a territory

if ( NoTerritories > 199999 ) {

static char errornum[ 20 ];

sprintf( errornum, "%d", NoTerritories );

L->Warn( "SkTerritories::PreProcessLandscape2(): ""Too many territories!", errornum );

exit( 1 );

}

Territories[ NoTerritories ] = new skTTerritory( x \* 10, y \* 10, range \* 10, qual, x, y, range ); // Here we have some variation in the quality requirements

Territories[ NoTerritories++ ]->TestNestPossibility();

// Build the per-territory lists of poygons and associated

// areas.

PreFillTerrPolyLists( Territories[ NoTerritories - 1 ] );

// Claim the territory in the grid

ClaimGrid( x, y, range );

x += range - 1;

range = 5;

continue;

}

// Not suitable too low quality, but can increase the area

bool done = false;

bool success = false;

do {

if ( ++range > 28 ) {

// Max. allowed territory size is 280m square.

done = true;

} else {

if ( IsExtGridPositionValid( x, y, range ) ) {

// Check Quality

// No point in rechecking the area already checked so just

// check the new area

qual += PreEvaluateHabitatStripX( x, y + range - 1, range );

qual += PreEvaluateHabitatStripY( x + range - 1, y, range );

//qual= PreEvaluateHabitat( x, y, range, range );

if ( qual >= targetqual ) {

// If so create it and claim the area on the grid

// Is OK so create a territory

if ( NoTerritories < 0 || NoTerritories > 199999 ) {

static char errornum[ 20 ];

sprintf( errornum, "%d", NoTerritories );

L->Warn( "SkTerritories::PreProcessLandscape2():"" Too many territories!", errornum );

exit( 0 );

}

success = true;

done = true;

Territories[ NoTerritories ] = new skTTerritory( x \* 10, y \* 10, range \* 10, qual, x, y, range );

Territories[ NoTerritories++ ]->TestNestPossibility();

// Build the per-territory lists of poygons and associated

// areas.

PreFillTerrPolyLists( Territories[ NoTerritories - 1 ] );

ClaimGrid( x, y, range );

x += range - 1;

range = 5;

}

}

}

} while ( !done );

if ( !success ) {

Grid[ x ] [ y ] = true;

if ( ++x >= m\_sim\_w\_div\_10 ) {

// Next line down

x = random( 3 );

if ( ++y >= m\_sim\_h\_div\_10 ) {

y = 0;

}

}

range = 5;

}

}

//l\_time = time( NULL );

//printf("%s\n", asctime(localtime(&l\_time)));

//printf("# territories: %d.\n", NoTerritories );

//DumpMapGraphics( "temp.ppm", L );

//exit(0);

}

|  |  |  |  |  |  |
| --- | --- | --- | --- | --- | --- |
| void SkTerritories::RemoveFemale | ( | int | *ref* | ) |  |

References skTTerritory::F\_Owner, and Territories.

Referenced by Skylark\_Female::OnMateDying(), Skylark\_Female::st\_Dying(), Skylark\_Female::st\_GivingUpTerritory(), and Skylark\_Female::st\_StoppingBreeding().

{

Territories[ ref ]->F\_Owner = NULL;

}

|  |  |  |  |  |  |
| --- | --- | --- | --- | --- | --- |
| void SkTerritories::RemoveMale | ( | int | *ref* | ) |  |

References skTTerritory::Owner, and Territories.

Referenced by Skylark\_Male::OnEvicted(), Skylark\_Male::OnMateLeaving(), Skylark\_Male::st\_AttractingAMate(), Skylark\_Male::st\_Dying(), and Skylark\_Male::st\_FollowingMate().

{

if ( ref != -1 ) Territories[ ref ]->Owner = NULL;

}

|  |  |  |  |  |  |
| --- | --- | --- | --- | --- | --- |
| void SkTerritories::Split | ( | int | *ref* | ) |  |

Duplicates the old territory.

Reduces the virtual size.

References skTTerritory::GetVirtualDiameter(), skTTerritory::m\_competitionscaler, NoTerritories, PreFillTerrPolyLists(), skTTerritory::SetNestPossibility(), skTTerritory::SetVirtualDiameter(), Territories, and CfgFloat::value().

Referenced by Skylark\_Male::ReEvaluateTerritory().

{

Territories[ NoTerritories++ ] = new skTTerritory( Territories[ ref]->m\_Location\_x, Territories[ ref]->m\_Location\_y,

Territories[ ref ]->Size, Territories[ ref ]->GetQuality(), Territories[ ref ]->m\_x\_div10, Territories[ ref ]->m\_y\_div10,

Territories[ ref ]->m\_range\_div10 );

PreFillTerrPolyLists( Territories[ NoTerritories - 1 ] );

Territories[ NoTerritories-1 ]->SetNestPossibility(Territories[ ref]->m\_nest\_valid,Territories[ ref]->m\_nest\_pos\_validx,Territories[ ref]->m\_nest\_pos\_validy);

Territories[ ref ]->m\_competitionscaler\*=cfg\_MaleSplitScale.value();

Territories[ NoTerritories-1 ]->m\_competitionscaler\*=cfg\_MaleSplitScale.value();

double Vdir=Territories[ ref]->GetVirtualDiameter()/1.41; // 1.41 is the theoretical diameter scale when dividing one territory into two

Territories[ ref]->SetVirtualDiameter(Vdir);

Territories[ NoTerritories-1 ]->SetVirtualDiameter(Vdir);

//printf("New %d\t%d\n",g\_land->SupplyDayInYear(),NoTerritories);

}

|  |  |  |  |  |  |
| --- | --- | --- | --- | --- | --- |
| Skylark\_Female \* SkTerritories::Supply\_F\_Owner | ( | int | *ref* | ) |  |

References skTTerritory::F\_Owner, and Territories.

Referenced by Skylark\_Female::st\_Finding\_Territory().

{

return Territories[ ref ]->F\_Owner;

}

|  |  |  |  |  |  |
| --- | --- | --- | --- | --- | --- |
| Skylark\_Male \* SkTerritories::Supply\_Owner | ( | int | *ref* | ) |  |

References skTTerritory::Owner, and Territories.

Referenced by Skylark\_Male::OnReHouse(), Skylark\_Female::st\_Finding\_Territory(), and Skylark\_Male::st\_FindingTerritory().

{

return Territories[ ref ]->Owner;

}

|  |  |  |  |  |  |
| --- | --- | --- | --- | --- | --- |
| int SkTerritories::Supply\_quality | ( | int | *ref* | ) |  |

References skTTerritory::GetQuality(), and Territories.

Referenced by Skylark\_Male::st\_FindingTerritory(), and Skylark\_Population\_Manager::TheSkylarkTerrsSupply\_quality().

{

return Territories[ ref ]->GetQuality();

}

|  |  |  |  |  |  |
| --- | --- | --- | --- | --- | --- |
| int SkTerritories::Supply\_size | ( | int | *ref* | ) |  |

References skTTerritory::Size, and Territories.

Referenced by Skylark\_Male::OnReHouse(), Skylark\_Male::st\_FindingTerritory(), and Skylark\_Population\_Manager::TheSkylarkTerrsSupply\_size().

{

return Territories[ ref ]->Size;

}

|  |  |  |  |  |  |
| --- | --- | --- | --- | --- | --- |
| skTTerritory \* SkTerritories::Supply\_terr | ( | int | *ref* | ) |  |

References Territories.

Referenced by Skylark\_Male::ConstructAHabitatTable(), Skylark\_Male::OnMateLeaving(), and Skylark\_Male::ReEvaluateTerritory().

{

return Territories[ ref ];

}

|  |  |  |  |  |  |
| --- | --- | --- | --- | --- | --- |
| int SkTerritories::Supply\_x | ( | int | *ref* | ) |  |

References skTTerritory::m\_Location\_x, and Territories.

Referenced by Skylark\_Male::OnReHouse(), Skylark\_Male::st\_FindingTerritory(), and Skylark\_Population\_Manager::TheSkylarkTerrsSupply\_x().

{

return Territories[ ref ]->m\_Location\_x;

}

|  |  |  |  |  |  |
| --- | --- | --- | --- | --- | --- |
| int SkTerritories::Supply\_y | ( | int | *ref* | ) |  |

References skTTerritory::m\_Location\_y, and Territories.

Referenced by Skylark\_Male::OnReHouse(), Skylark\_Male::st\_FindingTerritory(), and Skylark\_Population\_Manager::TheSkylarkTerrsSupply\_y().

{

return Territories[ ref ]->m\_Location\_y;

}

|  |  |  |  |  |  |
| --- | --- | --- | --- | --- | --- |
| bool SkTerritories::SupplyIsNestValid | ( | int | *ref* | ) |  |

inline

Referenced by Skylark\_Male::OnReHouse(), and Skylark\_Male::st\_FindingTerritory().

{

return Territories[ref]->m\_nest\_valid;

}

|  |  |  |  |  |  |
| --- | --- | --- | --- | --- | --- |
| Point SkTerritories::SupplyNestPosition | ( | int | *ref* | ) |  |

inline

References Point::m\_x, and Point::m\_y.

{

Point p;

p.m\_x = Territories[ref]->m\_nest\_pos\_validx;

p.m\_y = Territories[ref]->m\_nest\_pos\_validy;

return p;

}

|  |  |  |  |  |
| --- | --- | --- | --- | --- |
| int SkTerritories::SupplyNoFemaleOccupied | ( |  | ) |  |

References NoTerritories, and Territories.

{

int no=0;

for (int i=0; i<NoTerritories; i++) {

if (Territories[i]->F\_Owner != NULL) no++;

}

return no;

}

|  |  |  |  |  |
| --- | --- | --- | --- | --- |
| int SkTerritories::SupplyNoMaleOccupied | ( |  | ) |  |

References NoTerritories, and Territories.

{

int no=0;

for (int i=0; i<NoTerritories; i++) {

if (Territories[i]->Owner != NULL) no++;

}

return no;

}

|  |  |  |  |  |
| --- | --- | --- | --- | --- |
| int SkTerritories::SupplyNoTerritories | ( |  | ) |  |

References NoTerritories.

Referenced by Skylark\_Male::OnReHouse(), Skylark\_Female::st\_Finding\_Territory(), Skylark\_Male::st\_FindingTerritory(), and Skylark\_Population\_Manager::SupplyNoTerritories().

{

return NoTerritories;

}

|  |  |  |  |  |  |
| --- | --- | --- | --- | --- | --- |
| void SkTerritories::Tick | ( | void |  | ) |  |

inline

Referenced by Skylark\_Population\_Manager::DoFirst().

{

m\_qual\_cache\_filled = false;

}

|  |  |  |  |  |
| --- | --- | --- | --- | --- |
| void SkTerritories::UpdateQuality | ( |  | ) |  |

---

## Member Data Documentation

bool SkTerritories::Grid[1001][1001]

private

Referenced by ClaimGrid(), IsExtGridPositionValid(), IsGridPositionValid(), and PreProcessLandscape2().

int SkTerritories::m\_for\_iter\_x[1000]

private

Referenced by PreFillTerrPolyLists().

int SkTerritories::m\_for\_iter\_y[1000]

private

Referenced by PreFillTerrPolyLists().

int SkTerritories::m\_hash\_size

private

Referenced by PreFillQualCache(), and PreFillQualGrid().

int\* SkTerritories::m\_poly\_seen

private

Referenced by PreEvaluateQualGrid(), PreFillQualCache(), PreFillQualGrid(), PreFillTerrPolyLists(), and ~SkTerritories().

int\* SkTerritories::m\_poly\_size

private

Referenced by PreFillQualGrid(), PreFillTerrPolyLists(), and ~SkTerritories().

double\* SkTerritories::m\_qual\_cache

private

Referenced by PreCachePoly(), PreEvaluateQualGrid(), PreFillQualCache(), PreFillQualGrid(), and ~SkTerritories().

bool SkTerritories::m\_qual\_cache\_filled

private

Referenced by PreFillQualCache().

SkQualGrid\* \* SkTerritories::m\_qual\_grid

private

Referenced by PreEvaluateHabitat(), PreEvaluateHabitatStripX(), PreEvaluateHabitatStripY(), PreFillQualCache(), PreFillQualGrid(), PreFillTerrPolyLists(), and ~SkTerritories().

int SkTerritories::m\_qual\_grid\_signal

private

Referenced by PreFillQualGrid().

int SkTerritories::m\_sim\_h\_div\_10

private

Referenced by ClaimGrid(), IsExtGridPositionValid(), IsGridPositionValid(), PreEvaluateHabitat(), PreEvaluateHabitatStripY(), PreFillQualCache(), PreFillQualGrid(), PreFillTerrPolyLists(), PreProcessLandscape2(), and ~SkTerritories().

int SkTerritories::m\_sim\_w\_div\_10

private

Referenced by ClaimGrid(), IsExtGridPositionValid(), IsGridPositionValid(), PreEvaluateHabitat(), PreEvaluateHabitatStripX(), PreEvaluateHabitatStripY(), PreFillQualCache(), PreFillQualGrid(), PreFillTerrPolyLists(), PreProcessLandscape2(), and ~SkTerritories().

int SkTerritories::NoTerritories

private

Referenced by DumpMapGraphics(), EvaluateAllTerritories(), GetTerritoriesByDistance(), IsValid(), PreProcessLandscape2(), SkTerritories(), Split(), SupplyNoFemaleOccupied(), SupplyNoMaleOccupied(), SupplyNoTerritories(), and ~SkTerritories().

double SkTerritories::PolyHeightData[2500]

int SkTerritories::PolyRefData[2500]

Referenced by PreEvaluateQualGrid(), and PreFillTerrPolyLists().

int SkTerritories::PolySizeData[2500]

TTypesOfLandscapeElement SkTerritories::PolyTypeData[2500]

int SkTerritories::SimH

private

Referenced by PreFillQualGrid(), and SkTerritories().

int SkTerritories::SimW

private

Referenced by PreFillQualGrid(), and SkTerritories().

skTTerritory\* SkTerritories::Territories[400000]

private

Referenced by DumpMapGraphics(), EvaluateAllTerritories(), FemaleOccupy(), GetTerritoriesByDistance(), IsValid(), Occupy(), PreProcessLandscape2(), RemoveFemale(), RemoveMale(), Split(), Supply\_F\_Owner(), Supply\_Owner(), Supply\_quality(), Supply\_size(), Supply\_terr(), Supply\_x(), Supply\_y(), SupplyNoFemaleOccupied(), SupplyNoMaleOccupied(), and ~SkTerritories().

Landscape\* SkTerritories::TheLandscape

Referenced by PreEvaluateQualGrid(), PreFillQualGrid(), PrePoly2Qual(), PrePolyNQual(), and SkTerritories().

---

The documentation for this class was generated from the following files:

- skylarks\_all.h
- skylarks\_all.cpp


- SkTerritories
- Generated on Thu Jan 10 2013 13:15:37 for ALMaSS Skylark ODdox by
   1.8.1.1
